# Spectrum and Outcome of Prenatally Diagnosed Fetal Primary Cardiomyopathies—A Twenty-Year Overview

**DOI:** 10.3390/jcm12134366

**Published:** 2023-06-28

**Authors:** Adeline Walter, Elina Calite, Annegret Geipel, Brigitte Strizek, Florian Recker, Ulrike Herberg, Christoph Berg, Ulrich Gembruch

**Affiliations:** 1 Department of Obstetrics and Prenatal Medicine, University of Bonn, 53127 Bonn, Germany; elina.calite@gmail.com (E.C.); annegret.geipel@ukbonn.de (A.G.); brigitte.strizek@ukbonn.de (B.S.); florian.recker@ukbonn.de (F.R.); prof.berg@icloud.com (C.B.); ulrich.gembruch@ukbonn.de (U.G.); 2 Department of Pediatric Cardiology, University Hospital RWTH Aachen, 52074 Aachen, Germany; uherberg@ukaachen.de; 3 Division of Prenatal Medicine, Gynecological Ultrasound and Fetal Surgery, Department of Obstetrics and Gynecology, University of Cologne, 50937 Cologne, Germany

**Keywords:** primary fetal cardiomyopathy, cardiomyopathies, cardiomegaly, fetal echocardiography, prenatal diagnosis

## Abstract

Objective: to assess the course and outcome of fetuses affected by primary cardiomyopathy (CM). Methods: Retrospective study of 21 cases with prenatal diagnosis of a primary CM in one tertiary center over a period of 20 years. Charts were reviewed for echocardiographic findings, pregnancy outcome, and postnatal course. The utility of prenatal evaluation was discussed. Results: The mean gestational age (GA) at diagnosis was 26.7 (±5.1) weeks. A total of 33.3% (7/21) had associated anomalies. Genetic etiology was confirmed in 50.0% (10/20, with one case lost to follow up). The overall survival rate of the entire study population was 40% (8/20) including termination of pregnancy in 20% (4/20) and an intrauterine mortality rate of 5% (1/20). Of the initial survivors (*n* = 15), a neonatal and early infant mortality rate of 46.7% (7/15) was calculated. Prenatal isolated right ventricular involvement was the only identified significant parameter for survival (*p* = 0.035). Four phenotypical groups were identified: 42.9% (9/21) hypertrophic (HCM), 38.1% (8/21) dilated (DCM), 14.3% (3/21) isolated noncompaction (NCCM), and 4.8% (1/21) restrictive CM (RCM). Fetuses assigned to isolated NCCM revealed a 100% survival rate. Conclusion: Prenatal detection is feasible but needs to a introduce classification method for better consulting and management practices. A poor outcome is still observed in many cases, but an increase in examiners’ awareness may influence optimal multispecialized care.

## 1. Introduction

Primary cardiomyopathies (CMs) correspond to an important and heterogeneous group of myocardial disorders, which solely or predominantly target the myocardium and affect cardiac filling, contraction, or both [1]. Rarely seen prenatally, they occur as late onset anomalies and account for approximately 2–2.5% of all congenital heart diseases [2,3]. Fetal and neonatal outcomes are extremely poor, with a reported perinatal mortality rate of up to 50–82%, continuing into the neonatal period, with primary CMs being the most common cause of cardiac transplantation in childhood [4,5,6,7].

Fetal echocardiography remains the main diagnostic tool in prenatal diagnosis of primary CMs [7]. Based on clinical and anatomical presentation, categorization is most commonly performed into dilated (DCM), hypertrophic (HCM), and restrictive (RCM) cardiomyopathy and might be subdivided into isolated noncompaction cardiomyopathy (NCCM), as published data demonstrated its feasibility in prenatal echocardiographic detection [1,7,8,9,10,11]. Grouping different phenotypes is used to define the underlying cause, which is a significant determinant of neonatal outcome [5]. Clinical presentation, however, is highly variable and fetuses may have perinatal demise in almost one third of affected cases, or may present severe postnatal cardiac failure, or be born with a relatively benign neonatal course with a potential for recurrence [5,12]. Albeit unfavorable, echocardiographic predictors for an intrauterine demise have been defined; previously published data, limited to small study populations and heterogeneous cohorts, have failed to evaluate prognostic criteria for a tailored approach to intrauterine guidance on outcome [5,6,7,8].

With contradictory results, consulting and impact on management strategies has to be re-evaluated, especially as rapid increase in clinically available genetic testing facilitated confirmation of an identifiable genetic etiology in 40–80% of neonatal primary CMs, suggesting that not only the demand for prenatal counselling with a known family history will increase in significance, but also that suspecting prenatal primary CM should include targeted screening of the extended family and recurrence-risk counseling for subsequent pregnancies [7,13].

We provided an overview of fetuses diagnosed with primary CM at our center. Prenatal diagnosis was correlated for postnatal course and outcomes were compared to other published data. The utility of prenatal evaluation was discussed.

## 2. Materials and Methods

### 2.1. Patients

All cases with a prenatal diagnosis of fetal primary cardiomyopathy (PCM), detected in a 20 years period (2001 to 2021) in a tertiary referral center (University of Bonn, Bonn, Germany), were retrospectively reviewed for course and outcome.

### 2.2. Definitions

**Figure 1:**
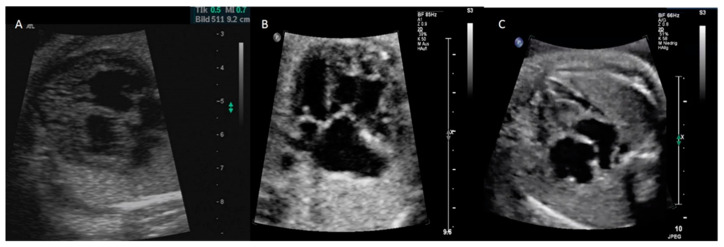
Different phenotypes of PCM illustrating HCM at 32 + 3 weeks gestation (**A**), isolated NCCM affecting only the right ventricle at 28 + 0 weeks (**B**), and RCM 34 + 5 (**C**).

Primary CM was defined according to the American Heart Association (AHA). Based on prenatal phenotype presentation, all cases were further divided into dilated (DCM), hypertrophic (HCM), restrictive (RCM), and isolated noncompaction (NCCM) cardiomyopathy (Figure 1) [1].

**Figure 2:**
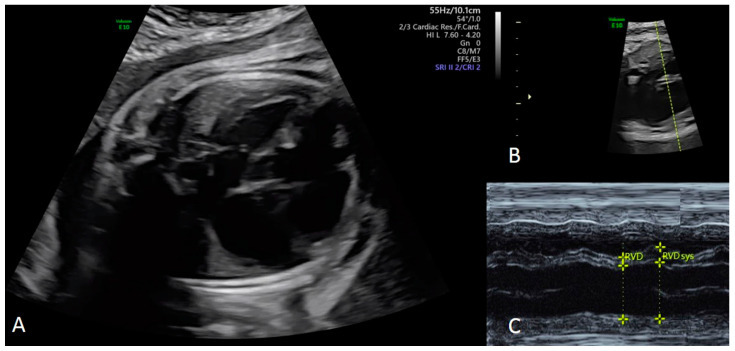
Dilative cardiomyopathy at the four chamber view in a fetus at 34 + 3 weeks gestation (**A**). Corresponding M-Mode cursor through the ventricles in an axial four chamber view of the fetal heart (**B**), with the corresponding M-Mode tracing (**C**). End-diastolic (EDD) (Z-score 3.48) and end-systolic (ESD) (Z-score 6.23) diameter of the right ventricle can be measured accurately to evaluate the shortening fraction (SF) [16]. EDD and ESD are demonstrated to be nearly the same with an SF of less than 5%. Changes in right ventricular diameters during the cardiac cycle are only caused by movement of the interventricular septum (IVS) (**C**).

In the presence of two different CM phenotypes, cases were assigned to the dominant subtype. DCM was diagnosed if one or both ventricles had qualitative dilation and there was qualitatively impaired systolic function (Figure 2) [14,15].

HCM was defined by the a presence of qualitative hypertrophy in one or both ventricles with an otherwise preserved systolic function [17,18]. Isolated NCCM was suspected in the appearance of an abnormal thick endocardial compact layer with prominent trabeculations best visualized at systole and deep intertrabecular recesses moving with the myocardium and being filled by direct blood flow from the ventricular cavity on color Doppler imaging. Ventricular function was unsuspicious [19]. RCM was defined as one or both atria enlarged compared to ventricles of normal or small size with hemodynamic alterations including a dominant E-wave and a very short-duration A-wave, pulsatile ductus venosus, and atrioventricular-valve regurgitation [1].

Cases in which CM was considered secondary to fetal structural heart diseases (e.g., twin-to-twin transfusion syndrome, maternal diabetes, viral infection, anemia, fetal supraventricular tachyarrhythmias, anti-SSA/anti-SSB antibodies, and for complete fetal heart block) were excluded.

### 2.3. Ultrasound Assessment

An anatomical survey and fetal echocardiography were performed in a standardized fashion, using a segmental approach with defined anatomical planes incorporating pulsed-wave and color Doppler imaging [20,21]. Multifrequent sector or curved array probes (5 MHz, 7.5 MHz or 9 MHz) were used for all ultrasound examinations (HDI IU22, Phillips, Hamburg, Germany; Voluson E8 and E10, GE Healthcare, Solingen, Germany). Data were retrieved from medical files and stored ultrasound images. Cardiomegaly was defined as a cardiothoracic diameter ratio (CTR) > 0.50 [22]. Cardiac and ventricular sphericity indexes, including ventricular basal and midventricular sphericity indexes, were calculated as described else were [23]. Fetal hydrops was diagnosed in the presence of a fluid accumulation in at least two compartments including polyhydramnios, ascites, generalized skin edema, and pericardial or pleural effusion. All cases included an evaluation of viral myocardial infections (including toxoplasmosis, coxsackievirus, echovirus, cytomegalovirus, and herpes simplex virus) and of maternal antibodies (anti-SSA and anti-SSB).

### 2.4. Outcome

Outcomes were categorized into five groups: termination of pregnancy (TOP), intrauterine fetal demise (IUFD), neonatal death (NND), death in infancy or childhood (ICHD), and survivors. Neonatal death was defined as death within the first 28 days of life. Postnatal assessment was collected from the neonates’ medical records and autopsy findings if available.

### 2.5. Data Analysis

Statistical analysis was performed using the Statistical Package for Social Sciences (SPSS 25.0, SPSS Inc., Chicago, IL, USA) statistical software. Intergroup comparison were made using one-way ANOVA with a post hoc test, Student’s *t*-test, or Fisher’s exact test. All values are given as mean ± standard deviation unless indicated otherwise. A *p* value of <0.05 was considered significant. All patients have given written informed consent for data collection, analysis, and their use for research, although the institutional review board of the University of Bonn does not require formal ethical approval for retrospective archived studies.

## 3. Results

**Figure 3:**
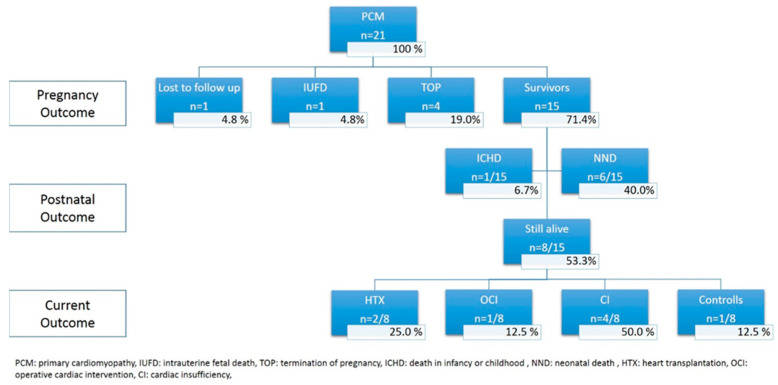
Flowchart summarizing the outcome of 21 fetuses prenatally diagnosed with primary CM.

During the study period, we identified 21 pregnancies available for analysis, including two patients with two pregnancies (Figure 3).

Mean maternal age was 31.6 years (±6.2) with a mean body mass index (BMI) of 26.8 kg/m^2^ (±5.5). In 19.0% (4/21) parental consanguinity and in 4.8% (1/21) maternal cardiac anomaly (Ebstein anomaly) was known. The gestational age at diagnosis was 26.7 weeks (±5.1), with none of the cases identified in the first trimester. A total of 11/21 (52.4%) fetuses were male. Suspicion of fetal heart disease accounted for 47.6% (10/21) of overall referral reasons. Other indications were family history of primary CM or known genetic mutations in 14.3% (3/21), hydrops fetalis in 14.3% (3/21), and in 23.8% (5/21) no cardiac specific reason. Biventricular involvement was the most common subtype (14/21; 66.7%). In the remaining 33.3% (7/21) predominant right ventricular dysfunction was observed as isolated tricuspid valve regurgitation with a preserved left ventricular function was seen on two-dimensional fetal echocardiography.

### 3.1. Prenatal CM Phenotype

In 42.9% (9/21) fetuses were assigned to HCM, in 38.1% (8/21) to DCM, in 14.3% (3/21) to isolated NCCM, and in 4.8% (1/21) to RCM. Prenatal cardiac function and echocardiographic characteristics are summarized in Table 1.

**Figure 4:**
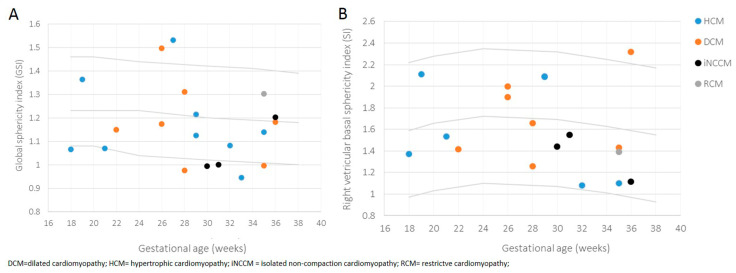
Scatterplot of global sphericity index (GSI) (**A**) and right ventricular basal sphericity index (SI) (**B**), according to gestational age of fetuses affected by primary CM, differentiated by provided phenotypes. Estimated 5th (lower), 50th (middle), and 95th (upper) centile curves are shown.

HCM and DCM were diagnosed at an earlier gestational age (24.2 weeks (±4.8) and 26.8 weeks (±3.8)) compared to isolated NCCM and RCM (31.2 weeks (±4.9) and 34.9 weeks), although not reaching significance (*p* = 0.080). Univentricular involvement varied significantly among the different phenotypes (*p* = 0.023), with the most univentricular involvement seen in the isolated NCCM group (66.6%). The global sphericity index (GSI) demonstrated a more globular cardiac shape in general with a mean GSI of 1.17 (±0.1) vs. 1.23 (50th centile) compared to published data by Crispi et al. (Figure 4 A) [23].

Fetuses assigned to the isolated NNCM phenotype revealed the most pronounced globular cardiac shape with the most pronounced globular right ventricular basal sphericity index (SI), although not reaching significance (*p* = 0.843) compared to other subtypes (Figure 4B). In all phenotypes the right basal ventricular sphericity index (SI) was lower compared to the left ventricular SI.

### 3.2. Additional Cardiac, Extracardiac and Genetic Anomalies

Seven fetuses (33.3%) showed additional anomalies, including two fetuses with a small ventricular septum defect, one fetus with a urogenital disorder, and one fetus of each with vermis hypoplasia, unilateral clubfoot, bilateral hydrothorax, and with lateral cervical cysts.

In total, nonchromosomal and chromosomal syndromes were diagnosed in 10/20 (50.0%) of fetuses (Barth syndrome (BTHS) (*Taffazin* gene mutation) in 2 fetuses; *MYBPC3* gene mutation in 2 fetuses; Noonan syndrome (*RIT1* gene mutation), LEOPARD syndrome (*PTPN11* mutation), Wolf-Hirschhorn syndrome (microdeletion 4p16.3), *KCNH* gene mutation, translocation (1;8) combined with duplication (1p 36.32) and deletion (Xp22.31,x1), and congenital Marfan syndrome in 1 fetus each), of which 60.0% (6/10) were assigned as HCM, and 40.0% (4/10) as DCM. Prenatal CM phenotypes in accordance with postnatal identified syndromes are shown in Table 2.

In one case, a mitochondrial myopathy was suspected but muscle biopsy revealed a normal result. Two further cases had an abnormality strongly suspicious for an underlying genetic etiology, resulting in an overall suspected genetic etiology in possibly as high as 60.0% (12/20). Baseline characteristics in comparison to neonatal outcome are displayed in Table 3.

Survivors at last follow up showed a significantly higher proportion of prenatal univentricular myocardial involvement (*p* = 0.035). Other investigated parameters revealed no significant difference, although there was a trend toward further malformations, hydrops fetalis, and a mitral valve regurgitation in the nonsurvivor group.

### 3.3. Outcome

The postnatal outcome with regard to different CM phenotypes are displayed in Table 4.

Figure 3 demonstrates the postnatal outcome of the entire study population. The overall mortality rate at last follow up was 60.0% (12/20, excluding one case lost to follow up. Intrauterine death occurred in one case (5.0%) with hydrops fetalis and severe systolic dysfunction of the right ventricle at 28 weeks; in this case, Barth syndrome was suspected, but postnatal histopathological examination revealed no specific result. In 4/20 cases (20.0%) parents opted for termination of three pregnancy, three of them due to a known genetic mutation and positive family history and one due to severe hydrops fetalis with global systolic cardiac dysfunction at 28 weeks. A total of 15 out of 20 (75.0%) fetuses were live born. In six of the fifteen initial survivors (40%) neonatal death occurred. The mean gestational age within this group at delivery was 32.8 weeks (±4.01). In one case, Uhl’s anomaly with severe right ventricular dysfunction including severe tricuspid regurgitation and decreased cardiac output was prenatally seen. Cardiac failure could not be stabilized and NND occurred on the fifth day of life. In one case, left ventricular dysfunction was initially dominant, although prenatally biventricular involvement (RCM) was diagnosed. Multiorgan failure with sepsis led to NND on the 15th day of life. In the remaining four neonates, genetic abnormalities were known (Barth, Noonan, LEOPARD, and Marfan syndrome). Terminal cardiac failure lead to death on the 17th, 12th, 5th, and 10th day of life, respectively. In one neonate being affected by Barth’s syndrome, death occurred at an age of three years. As progressive cardiac failure and sepsis due to pneumonia occurred, the parents denied listing for cardiac transplantation and opted for palliative care.

Eight out of fifteen neonates are still alive. In two cases, cardiac transplantation was needed, due to progressive cardiac failure. In one neonate the prenatal diagnosis of a NCCM was changed into small vessel disease via histopathological examination. In the other neonate, long-QT syndrome was detected. Cardiac transplantation was performed at the age of 4 months and of 4 years, respectively.

In two neonates (13.3%; 2/15) phenotypical classification changed. Both were prenatally assigned to NCCM. One of them had a complete recurrence at the age of 8 years. In the other case initial right ventricular hypertrophy and dilation was observed. At the age of 12 years, the right ventricle was unsuspicious; however, the patient is now suffering from a dysplastic aortic valve and short stature. Genetic etiology is highly suspicious as first degree relatives are also suffering from a non-classified heart disease. In one further case, HCM led to severe stenosis of the pulmonary valve, so balloon valvuloplasty was performed at an age of 6 months. In addition, a premature craniosynostosis was seen and surgical procedure was carried out. Although the findings were highly suspicious for Noonan syndrome, no genetic testing was performed until now. In the remaining two out of three cases, Uhl’s anomaly was diagnosed. Postnatal genetic evaluation lead to the diagnosis of a genetic mutation in one case with the result of translocation (1;8), combined with duplication (1p 36.32) and deletion (Xp22.31,x1). Both patient are being treated for cardiac failure. In the last case no further cytogenetic description of myopathy was diagnosed, as suspected Pompe disease could not be confirmed via muscle biopsy.

## 4. Discussion

Fetal cardiomyopathies carry a substantial burden of disease due to the risk of morbidity and mortality and a missing curative therapy. With a still-undefined incidence in prenatal series varying from 0.004% to 7%, compared to 0.001% in our cohort, data remains limited and prenatal diagnosis requires a high level of clinical suspicion [5,6,7].

### 4.1. Categorization System

In retrospective studies, primary CMs were categorized either as hypertrophic or nonhypertrophic/dilated phenotypes, with some further differentiating a mixed phenotype, suggesting that a simplifying classification appears to be more accurate and reproducible [5,6,8]. However, increased sophistication in ultrasound technologies has proposed a more detailed classification encompassing RCM and isolated NCCM, taking varying prognostic parameters and adapted prenatal genetic testing into account [7,24,25]. Consequently, in the absence of standardized guidelines and multiple definitions in use, the distribution of fetal phenotypes remains unclear with a prevalence of HCM varying from 18.0% to 60.0% and of DCM from 11.0% to 72.0%, as the most common subgroups [5,6,8]. In accordance to published data, we were able to identify HCM (42.9%) and DCM (38.1%), as the main phenotypes and further differentiated isolated NCCM in 14.3% and RCM as the rarest phenotype. Comparing results for NCCM, the study by Trakmulkichkarn et al., revealed a higher proportion of NCCM (26.0%) [7]. This difference might be explained by the small number of cases in our study, focusing only on the isolated uni-/biventricular NCCM subtype, as well as the recent increase in clinical awareness, leading to the diagnosis of all three cases in a later era. Further, with myocardial compaction occurring to a greater extent in the LV myocardium than in the RV myocardium, it remains difficult to distinguish normal variants of physiologically more trabeculated RV from pathological isolated NCCM, causing a potential underestimation. Data regarding this specific phenotype must, therefore, be treated with caution.

### 4.2. Echocardiographic Evaluation

**Figure 5:**
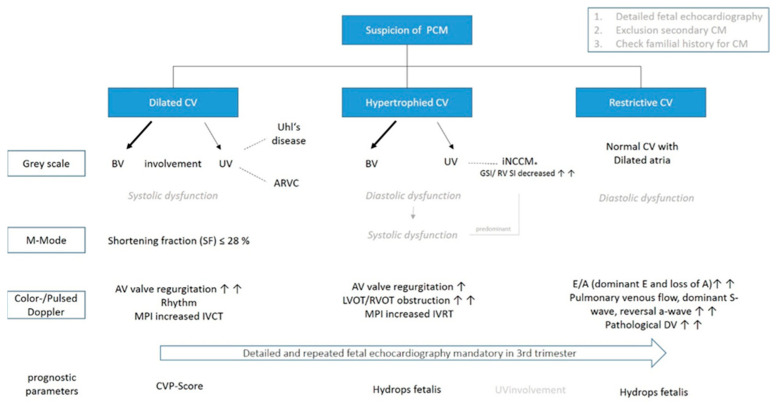
Flowchart illustrating a possible fetal echocardiographic examination in cases of suspected PCM. Mixed phenotypes and new echocardiographic techniques are not listed for better orientation. Arrows represent frequent occurrence (AV = artioventricular; ARVC = Arrhythmogenic right ventricular cardiomyopathy; CV = cardiac ventricles; CVPS = cardiovascular profile score; DV = ductus venosus; GSI = global sphericity index; RV SI = right ventricular basal sphericity index; MPI = myocardial performance index; IVCT = isovolumetric contraction time; IVRT = isovolumetric relaxation time; iNCCM* = may affect also both ventricles or occur with the other phenotypes).

Focusing on prenatal echocardiographic predictive parameters, neither the cardiovascular profile score nor the Tei index seem to be able to reliably predict outcomes dealing with primary CM in general, although it might be applied for DCM [6]. Referring to RCM, postnatal research demonstrated an elevated pulmonary vascular resistance, PR prolongation, and the elevation of mitral valve Doppler E/e’ ratio being associated with increased mortality, leaving the utility for the prenatal course unproven [26,27]. Regarding NCCM, postnatal data have shown a strong relationship between cardiac phenotype and risk of death or transplantation distinguishing isolated phenotype of NCCM from hypertrophic, dilated, and restrictive, mandating further prenatal research potentially using fetal MRI, as prognostic parameters might be found [28,29]. A possible algorithm for prenatal evaluation in cases of suspicion of a primary CM is demonstrated in Figure 5.

In our study, we identified uni-right ventricular involvement as the only significant parameter for survival (*p* = 0.035), with the most proportion seen in the isolated NCCM phenotype (*p* = 0.023), which might be explained as right ventricular function has the chance to improve after birth [15]. With decreasing postnatal right ventricular afterload, the diseased right side of the heart undergoes physiological unloading and might recover cardiac function. Because in fetal life the right heart is exposed to a greater workload than the left heart, functional analysis of the right ventricle in utero can underestimate its pumping capacity and vice versa. Consequently, biventricular cardiomyopathy may occur prenatally with isolated right ventricular dysfunction and may only be detected after birth. For prenatal evaluation, changed myocardial maturation during gestation and the altered hemodynamic situation from prenatal to postnatal course must, therefore, be taken into account [15].

### 4.3. Genetic

We observed a genetic etiology in 50.0% (10/20) of patients and suspected it in 60.0% (12/20), which seems to be higher than previously reported [7]. The difference might be explained by including two patients with two pregnancies, with a known gene mutation, but also might be explained by the increasing use of genetic analysis, which will likely further increase the number. However, the interpretation of genetic results needs to be thoughtful, as clinical severity may vary with age for different morphological manifestations of same gene mutation and not all variants identified via genetic testing will be clinically significant or disease-causing [30]. Moreover, Sun et al. identified a distinct genetic spectrum among NCCM in fetal, pediatric, and adult patients, mandating a possible need for different molecular genetic testing/panel and leaving it uncertain whether insights obtained from pediatrics and adult patients can be transferred to fetal primary CMs and vice versa [30,31,32]. Despite this controversy, there are still several advantages for detailed genetic evaluation, even in the occurrence of an intrauterine fetal demise: (1). Some mutations (*MYH7* gene) have been associated with structural congenital heart disease as well as CM. (2). The outcome and risk for recurrence or subsequent pregnancies might be evaluated. (3). CM may be diagnosed in previously undiagnosed familial members, as demonstrated in our study by identifying a same gene mutation of affected fetuses in 20% (3/15) of the parents.

### 4.4. Outcome

In a retrospective study, a working group of Toronto found perinatal survival rates of only 18% for fetuses with DCM and 48% for HCM, evaluating CMs in general [8]. In 2014, the same group found improved outcomes in fetuses with dilated compared to hypertrophic CM (45 vs. 47%) [5]. Improvement in outcome was assigned to a better treatment of antibody-mediated CMs [5]. However, nearly 20% of the study population had to be classified as secondary CMs, biasing outcomes [1,5]. Recently published data identified an overall survival rate of 32% (12/38) with a given intrauterine mortality rate of 50% (19/38) [7]. The neonatal and early infant mortality rate was 37% (7/19) [6]. The mortality rate was found to be 71% in those with DCM and 50% in those with HCM [7]. In our study, the overall survival rate was 40.0%, comparable to data by Trakmulkichkarn et al. [7]. The mortality rate was found to be 62.5% (5/8) for DCM and 75.0% for HCM (6/8). The difference in a better survival rate for DCM might be explained by modern perinatal and postnatal management strategies, including improved resuscitation, the growing scope and use of ventricular assist devices, and option for cardiac transplantation. The cardiac transplantation rate of 13.3% was significantly higher compared to previously published data (3.2–6.5%) [5,7,33]. However, as pediatric data provide a similar transplantation rate in the recent era, the earlier recognition of heart failure and medical management seems more likely to cause an improve in outcome [33]. Higher mortality rates of HCM might be explained by a poorer survival in those with a genetic etiology, as extensive life-sustaining interventions might not be offered or opted in to in the context of expected extracardiac manifestations of the underlying disorder. Genetic etiology was known in all cases with neonatal or early infant death assigned to HCM, and 71.4% (5/7) in general, which was higher compared to previous published data with a confirmed genetic etiology in 57% of fetuses assigned to HCM [7]. With a survival rate of 100% (3/3) at last follow up referring to NCCM in our study, the outcome was better compared to reported data on 43% being life born [28]. The difference is highly explained due to the small case load in our study, as well as the missing consensus on the diagnostic criteria and classification of NCCM and the possibility of physiological myocardial maturation being mistaken [19,28,32].

### 4.5. Limitations

This study has some limitations. The sample size is relatively small, with a particularly limited case number for subgroup analysis of the different CM phenotypes. Intergroup statistical comparisons are, therefore, of relatively limited value. Given the retrospective study design, genetic studies were not available for all fetuses. In addition, no standardized protocol was used, so clinical and echocardiographic evaluated data were not available from all patients. Furthermore, due to being performed at a single institution, a demographic bias cannot be excluded.

## 5. Conclusions

Prenatal detection of primary CM is desirable as it might change the management and outcome in affected patients. If prenatally suspected, screening and genetic evaluation of other family members should be performed, as primary CM is highly hereditable, resulting in a high risk for recurrence in subsequent pregnancies. In case of a known familial risk, serial monitoring is warranted with evaluations of cardiac function. Although prenatal predictive parameters remain limited, evaluation of ventricular involvement might be seen as a prognostic parameter for survival. Delivery should take place in a perinatal center with multispecialized care.

Nevertheless, future research with a collection of cases to assess outcomes and the impact of management strategies is required and risk factors for adverse outcomes to assist in risk stratification, parental counselling, and appropriated resources at delivery need to be defined.

## Figures and Tables

**Table 1:** Cardiac function and echocardiographic characteristics of the different CM phenotypes.

Parameter	Over all *n* = 21	DCM *n* = 8	HCM *n* = 9	Isolated NCCM *n* = 3	RCM *n* = 1	*p*-Value
Fetal hydrops	9	3	6	0	0	0.141
Arrhythmia	2	1	1	0	0	0.912
FHR	21	146.4 ± 13.4	132.3 ± 8.0	139.7± 10.8	137	0.190
EFE	1	1	0	0	0	0.636
TI	13	5	4	3	1	0.234
TI Vmax m/s	1.4 ± 1.4	2.4 ± 1.0	2.4 ± 1.2	1	2.1	0.276
MI	6	1	4	0	1	0.121
CTR	0.55 ± 0.1	0.62 ± 0.1	0.48 ± 0.1	0.57 ± 0.1	0.51	0.323
DV REDF	6	3	2	0	1	0.645
Pulsatile UV flow	7	2	2	2	1	0.329
Associated anomalies	7	2	5	0	0	0.496
Uni-/Bi-ventricular involvement	7/14	5/3	0/9	2/1	0/1	0.023
GSI (LCD/TCD)	1.17 ± 0.10	1.16 ± 0.13	1.12 ± 0.12	1.06 ± 0.12	1.3	0.579
LVLD/LVMTD	2.55 ± 0.96	2.21 ± 0.53	2.64 ± 1.23	2.88 ± 1.17	3.54	0.509
RVLD/RVMTD	2.23 ± 0.96	2.05 ± 0.67	2.63 ± 1.26	1.51 ± 0.22	2.55	0.487
LVLD/LVBD	1.81 ± 0.46	1.72 ± 0.22	1.74 ± 0.57	2.19 ± 0.68	1.91	0.497
RVLD/RVBD	1.66 ± 0.66	1.74 ± 0.36	1.71 ± 0.98	1.37 ± 0.23	1.39	0.843

Abbreviations (in alphabetical order): CTR = cardiothoracic diameter ratio; DV = ductus venosus; DCM = dilated cardiomyopathy; EFE = endocardial fibroelastosis; FHR = fetal heart rate; GSI = global sphericity index; HCM = hypertrophic cardiomyopathy; LCD = longitudinal cardiac diameter; LVBD = left ventricular basal diameter; LVLD = left ventricular longitudinal diameter; LVMTD = left ventricular midtransverse diameter; MI = mitral valve insufficiency, NCCM = noncompaction cardiomyopathy; REDF = reversed end-diastolic flow; RCM = restrictive cardiomyopathy; RVBD = right ventricular basal diameter; RVLD = right ventricular longitudinal diameter; RVMTD = right ventricular midtransverse diameter; TCD = transverse cardiac diameter; TI = tricuspid insufficiency, UV = umbilical vein.

**Table 2:** CM Phenotypes in accordance with postnatal syndromes.

DCM *n* = 8		HCM *n* = 9		NCCM *n* = 3		RCM *n* = 1	
*n*		*n*		*n*		*n*	
1	Marfan syndrome	2	Barth syndrome	3	none identified	1	none identified
1	Wolf-Hirschhorn syndrome	1	Noonan syndrome				
3	Uhl’s anomaly	1	LEOPARD syndrome				
1	Long-QT syndrome	2	*MYBPC3* gene mutation				
2	None identified	3	None identified				

Abbreviations (in alphabetical order): DCM = dilated cardiomyopathy; HCM = hypertrophic cardiomyopathy; NCCM = non-compaction cardiomyopathy; RCM = restrictive cardiomyopathy.

**Table 3:** Comparison neonatal outcome with respect to baseline characteristics.

Parameter	Total (*n* = 15)	Alive (*n* = 8)	Death (*n* = 7)	*p*-Value
GA at delivery (wks)	35.0 (±3.6)	36.4 (±3.6)	33.8 (±3.4)	0.176
Birth weight (g)	2325.3 (±807.8)	2652.1(±778.4)	2039.2 (±765.2)	0.149
Gender (f/m)	6/9	4/3	2/6	0.315
Univentricular involvement	7	6	1	0.035
Associated anomalies	6	2	4	0.378
Hydrops fetalis	7	2	5	0.214
MI	3	0	3	0.200
TI	9	5	4	0.378
TI vmax m/s	2.0 (±1.2)	1.5 (±0.6)	2.5 (±0.5)	0.207

Abbreviations (in alphabetical order): F = female; GA = gestational age; M = male; MI = mitral valve insufficiency; TI = tricuspid valve insufficiency.

**Table 4:** Postnatal outcome of the different CM phenotypes.

	Total (%)	IUFD	TOP	ICHD	NND	HTX	Still Alive
HCM	9 (42.9)	0	2	1	3	0	2 *
DCM	8 (38.1)	1	2	0	2	1	3
NCCM	3 (14.3)	0	0	0	0	1	3
RCM	1 (4.8)	0	0	0	1	0	0

Abbreviations (in alphabetical order): DCM = dilated cardiomyopathy; HCM = hypertrophic cardiomyopathy; HTX = heart transplantation; ICHD = infancy or childhood death; IUFD = intrauterine fetal demise; NCCM = non-compaction cardiomyopathy; NND = neonatal death; RCM = restrictive cardiomyopathy; TOP = termination of pregnancy; * one case with lost to follow up excluded.

## Data Availability

The data presented in this study are available on request from the corresponding author.
